# Microencapsulation of Plant Phenolic Extracts Using Complex Coacervation Incorporated in Ultrafiltered Cheese Against AlCl_3_-Induced Neuroinflammation in Rats

**DOI:** 10.3389/fnut.2022.929977

**Published:** 2022-06-29

**Authors:** Tarek N. Soliman, Dina Mostafa Mohammed, Tamer M. El-Messery, Mostafa Elaaser, Ahmed A. Zaky, Jong-Bang Eun, Jae-Han Shim, Marwa M. El-Said

**Affiliations:** ^1^Dairy Department, Food Industries and Nutrition Research Institute, National Research Centre, Cairo, Egypt; ^2^Department of Nutrition and Food Sciences, Food Industries and Nutrition Research Institute, National Research Centre, Cairo, Egypt; ^3^Department of Food Technology, Food Industries and Nutrition Research Institute, National Research Centre, Cairo, Egypt; ^4^Department of Food Science and Technology, Chonnam National University, Gwangju, South Korea; ^5^Natural Products Chemistry Laboratory, Biotechnology Research Institute, Chonnam National University, Gwangju, South Korea

**Keywords:** red beet, broccoli, spinach leaves, phenolic extract, UF-cheese, complex coacervation, antioxidant enzymes, neuroinflammation

## Abstract

Plant-derived phenolic compounds have numerous biological effects, including antioxidant, anti-inflammatory, and neuroprotective effects. However, their application is limited because they are degraded under environmental conditions. The aim of this study was to microencapsulate plant phenolic extracts using a complex coacervation method to mitigate this problem. Red beet (RB), broccoli (BR), and spinach leaf (SL) phenolic extracts were encapsulated by complex coacervation. The characteristics of complex coacervates [zeta potential, encapsulation efficiency (EE), FTIR, and morphology] were evaluated. The RB, BR, and SL complex coacervates were incorporated into an ultrafiltered (UF) cheese system. The chemical properties, pH, texture profile, microstructure, and sensory properties of UF cheese with coacervates were determined. In total, 54 male Sprague–Dawley rats were used, among which 48 rats were administered an oral dose of AlCl_3_ (100 mg/kg body weight/d). Nutritional and biochemical parameters, including malondialdehyde, superoxide dismutase, catalase, reduced glutathione, nitric oxide, acetylcholinesterase, butyrylcholinesterase, dopamine, 5-hydroxytryptamine, brain-derived neurotrophic factor, and glial fibrillary acidic protein, were assessed. The RB, BR, and SL phenolic extracts were successfully encapsulated. The RB, BR, and SL complex coacervates had no impact on the chemical composition of UF cheese. The structure of the RB, BR, and SL complex coacervates in UF cheese was the most stable. The hardness of UF cheese was progressively enhanced by using the RB, BR, and SL complex coacervates. The sensory characteristics of the UF cheese samples achieved good scores and were viable for inclusion in food systems. Additionally, these microcapsules improved metabolic strategies and neurobehavioral systems and enhanced the protein biosynthesis of rat brains. Both forms failed to induce any severe side effects in any experimental group. It can be concluded that the microencapsulation of plant phenolic extracts using a complex coacervation technique protected rats against AlCl3-induced neuroinflammation. This finding might be of interest to food producers and researchers aiming to deliver natural bioactive compounds in the most acceptable manner (i.e., food).

## Introduction

Recently, plants have garnered considerable research attention. They contain metabolites and compounds such as phenolics, flavonoids, alkaloids, anthocyanins, glycosides, and peptides. These compounds have a number of pharmacological effects, including immunomodulatory, antinociceptive, anti-inflammatory, antioxidant, antibacterial, anticarcinogenic, antiulcer, gastroprotective, antifungal, antispasmodic, antiviral, aphrodisiac, emergency contraception, hepatoprotective, antihyperglycemic, antilipidemic, nephroprotective, and antiamnesic effects (1–3).

However, these compounds become unstable under oxidation, light, heating, and moisture (4). Furthermore, the usefulness of bioactive constituents is inextricably linked to their bioavailability, which means that they must be properly digested in the stomach and then transported through the blood before reaching the target cells. However, they may be destroyed by pH changes in the gastrointestinal tract (5).

Microencapsulation technology is used to maintain delicate substances in harsh environmental conditions. This technology can also facilitate regulated release and disguise undesirable sensory characteristics of certain substances (6, 7). Encapsulation using complex coacervation is an approach related to the interaction of two oppositely charged particles in the aqueous phase, in which an interaction between the shell components (typically proteins and carbohydrates) takes place around the bioactive compounds (8). These coacervates are used to encapsulate different products, such as vitamins, sugars, and phenolics (9–12).

Many recent studies have illustrated the relevance of cheese as an excellent source of critical nutrients for the human body. As a result, adding cheese to the food system may encourage the consumption of herbs, fruits, and vegetables compared to diets not including cheese (13, 14).

Combining the aforementioned elements with cheese has garnered substantial attention in human health and disease control research by supplying health-promoting aspects and increasing overall nutrient consumption, thereby improving diet quality. Aluminum (Al) is a neurotoxic chemical that has been found in contaminated food and water and is also subject to particle inhalation by people in certain environments and experimental animals (15–17). Aluminum has been linked to the pathologic progression of a variety of brain diseases and was reported to be among the factors that cause neuroinflammation and deficits in cognitive functions. Neuroinflammation alters the density of dendritic spines, thereby contributing to cognitive impairment and neurodegenerative diseases (18, 19). Numerous *in vitro*, experimental, and human investigations have shown that Al leads to oxidative stress in the brain (20, 21). Manufacturing reactive oxygen species deplete antioxidant systems and promote lipid peroxidation, mutagenesis, and protein alteration, along with other issues (22, 23). Moreover, Al causes impairment in long-term memory (24) and hippocampal long-term potentiation (25). The verification of neuroprotective therapies is of particular importance, given that there are currently no cures for neurodegenerative diseases (26).

To our knowledge, no research has used encapsulated phenolic extracts [spinach leaf (SL), broccoli (BR), and red beet (RB)] for neuroinflammation, particularly their integration into UF cheese. Furthermore, phenolic extracts and cheese are good delivery vehicles for bioactive compounds, particularly phenolic compounds. As a result, this study integrated encapsulation, phenolic extracts, and UF cheese to preserve key bioactive components and also investigated the effects of these extracts on experimental rats against AlCl_3_-induced neuroinflammation.

## Materials and Methods

### Materials

The Animal Production Research Institute, Agriculture Research Center, Dokki, Egypt, supplied fresh ultrafiltration buffalo cream retentate. Gaglio Star (Spain) provided the fungal rennet powder (RENIPLUS) derived from Mucor miehei. RB (*Beta vulgaris*), BR (*Brassica oleracea var. italica*), and SL (*Spinach oleracea*) were acquired in a vegetable market in Giza. The analytical- and laboratory-grade chemicals and solvents utilized in the investigation were purchased from Sigma chemicals company (St. Louis, United States).

### Methods

#### Extraction of Phenolic Compounds

RB, BR, and SL were chosen for lack of flaws and then cleaned under flowing water. A measure of 10 g of each plant sample was added to 200 mL of ethanol (80%) and placed in an ultrasonicator for 1 min at room temperature. The samples were centrifuged, and the supernatant was separated. The extraction procedure was conducted three times. The solvent was evaporated by using a rotary evaporator (Büchi R20, Switzerland), and the residue was powdered using a freeze dryer (Labconco cooperation, Kansas City, United States) at –52°C for 48 h under 0.1 mPa and stored at –18°C (27).

#### High-Performance Liquid Chromatography

An Agilent 1260 series was used for high-performance liquid chromatography (HPLC) analysis. An Eclipse C18 column (4.6250 mm i.d., 5 m) was utilized for the separation. At a flow rate of 1 mL min G1, the mobile phase was composed of water (A) and 0.05% trifluoroacetic acid in acetonitrile (B). The mobile phase was set in the following order: 0 min (82% A), 0–5 min (80% A), 5–8 min (60% A), 8–12 min (60% A), 12–15 min (85% A), and 15–16 min (82% A). The multiwavelength detector was monitored at 280 nm. For each of the sample solutions, the injection volume was 10 L. The temperature in the column was kept constant at 35°C.

#### Preparation of Complex Coacervate Microcapsules

Cilek et al. (28) and El-Messery et al. (29) clarified that microcapsules of RB, BR, and SL powdered extracts can be prepared using a complex coacervation technique utilizing gum arabic (GA) and whey protein concentrate (WPC). We initially dissolved 3% (w/w) WPC in distilled water (40°C) once it became a homogeneous mixture. Then, we dissolved 1% (w/w) GA in distilled water at 25°C. The microcapsules were prepared by dissolving the powdered extract in the WPC solution at a ratio of 1:10, followed by diluting three to four times with distilled water at 50°C. The previous mixture (powdered extract and WPC) was mixed with the GA solution and centrifuged at 800 rpm. To generate electrostatic contact between WPC and GA, the pH of this combination was changed to 3.75 by infusion of 1% citric acid (added dropwise). The microencapsulation strategy was conducted at 25°C and then cooled to 5°C h^–1^. Last, the resultant complex was powdered using a freeze dryer (Labconco cooperation, Kansas City, United States) at –52°C for 48 h under 0.1 mPa.

#### Characterization of Microcapsules

##### ζ-Potential

To assess zeta potential, a dynamic light scattering instrument (Nano ZS, Malvern Instruments, Worcestershire, United Kingdom) was employed.

##### Total Phenolic Content

The total phenolic content (TPC) of the samples was evaluated according to the technique described in Zaky et al. (30). Folin–Ciocalteu reagent (100 μL) was used, followed by the addition of 1.58 mL of DW, and 20 μL of the sample. After 3 min, 300 μL of Na2CO3 (20%) was added. The mixture was left to stand at room temperature for 30 min. Then, the absorbance was estimated at 765 nm using a spectrophotometer (Cary 60 UV–Vis, Agilent Technologies, United States). The findings are presented as milligrams of gallic acid equivalent per gram.

##### Surface Phenolic Content

The Surface Phenolic Content (SPC) of the complex coacervates was determined according to the method described by Saénz et al. (31). A total of 100 mg of microcapsules was dispersed in 1 mL of ethanol–methanol mixture (1:1, *v/v*) for 1 min. The amounts of surface phenolic compounds were measured and quantified using the same method described in the TPC section.

##### Encapsulation Efficiency

The following equation (32) was used to calculate the EE of microcapsules:


E⁢E=(TPC-SPC)/TPC×100

##### Morphology

A scanning electron microscope (Quanta FEG 250 SEM) (Thermo Fisher Scientific, Oregon, United States) was used to characterize the particle structure of RB, BR, and SL microcapsules.

##### Fourier Transform Infrared Spectroscopy

Hu et al. (33) suggested a technique to identify chemical structures using an fourier transform infrared spectroscopy (FT-IR) spectrophotometer (Nicolet iS10, Thermo Fisher Scientific Co., Ltd., Waltham, Massachusetts, United States). In a ceramic mortar, the powdered sample was mixed with KBr powder and crushed into pellets. The FT-IR spectrum of the sample was obtained at a frequency of 4 cm^–1^ in the transmission mode in a wavelength range of 500–4,000 cm^–1^.

##### Preparation of Ultrafiltration Soft Cheese

UF cheese was prepared as per the method of Hala et al. (13). Fresh UF full cream retentate was used to make UF soft cheese, which was pasteurized at 72°C for 15 s, cooled and adjusted to 42°C, and then split into four batches. The first batch served as a control, and the other three batches were mixed separately with the RB, BR, and SL complex coacervates (equivalent to 100 mg of phenolic content in each microcapsule). Rennet was added and packed in plastic cups (100 mL) and then incubated (42°C) until full coagulation (40 min). The cheese samples were stored at a low (5 ± 2°C) temperature. Three duplicates from separate batches were prepared and examined.

##### Chemical Analysis

The chemical properties of UF cheese formulations were evaluated as described in AOAC (34). A pH meter (Jenway 3510) was utilized to determine the pH of the UF cheese samples.

##### Texture Profile Analysis

The parameters of texture assessment (cohesion, hardness, springiness, gumminess, and chewiness) for UF cheese samples were examined utilizing the dual stress test (TMS-Pro Texture Analyzer, United States) (35).

##### Sensory Evaluation

As indicated by Clark et al. (36), the UF cheese samples were analyzed for sensory qualities at 5 ± 2°C. A total of 10 panelists from the Dairy Department, National Research Centre, assessed the cheese samples for color and appearance, body and texture, and flavor (50, 40, and 10, respectively).

##### Experimental Animals

A total of 54 male Sprague–Dawley rats between 7 and 9 weeks of age and weighing 200–250 g were obtained from the National Care Unit, NRC, Cairo, Egypt. The animals were fed a basic balanced diet and water for 7 days before the study to enable acclimatization and ensure normal development and behavior. Individualized solid bottom cages were used to acclimate the rats in a temperature-regulated (23°C) environment at 40–60 g/100 g relative moisture, were lighted artificially (12 h dark/light cycle), and were free of contaminants. All rats were handled in accordance with the Animal Experimental Guidelines approved by the Ethical Committee of Medical Research, National Research Centre, Egypt, and the National Legislation on Lab Animal Rescue and Usage.

##### Diet Formulation

The basic balanced diet consisted of casein (150 g/kg), unsaturated fat (100 g/kg), sucrose (220 g/kg), maize starch (440 g/kg), cellulose (40 g/kg), salt mixture (40 g/kg), and vitamin mixture (10 g/kg) (37, 38). The salt and vitamin mixture was created using the AIN-93 M diet as a guide (39).

##### Induction of AlCl_3_ Neuroinflammation

AlCl_3_ solution was prepared for oral administration. AlCl_3_ was solubilized in distilled water and given orally at a dosage of 100 mg/kg body weight [BW]/d) (1 mL/rat/d for 4 weeks) (40).

##### Experimental Design

This study used 54 rats divided into nine groups (six rats each) described as follows:

• **Negative control:** Normal rats fed a basic balanced diet.
• **Positive control:** Rats with AlCl_3_-induced neuroinflammation were orally administered anhydrous AlCl_3_ daily for 4 weeks.
• **Group (1):** Rats with AlCl_3_-induced neuroinflammation were fed daily and orally administered the RB complex coacervate for 4 weeks (300 mg/kg BW/d) (41) 1 h before AlCl_3_ induction.
• **Group (2):** Rats with AlCl_3_-induced neuroinflammation were fed daily and orally administered the BR complex coacervate for 4 weeks (1.5 g/kg BW/d) (42) 1 h before AlCl_3_ induction.
• **Group (3):** Rats with AlCl_3_-induced neuroinflammation were fed daily and orally administered the SL complex coacervate for 4 weeks (400 mg/kg BW/d) (43) 1 h before AlCl_3_ induction.
• **Group (4):** Rats with AlCl_3_-induced neuroinflammation were fed UF cheese for 4 weeks 1 h before AlCl_3_ induction.
• **Group (5):** Rats with AlCl_3_-induced neuroinflammation were fed UF cheese supplemented with the RB complex coacervate (45 mg/2.25 g UF/rat/d) for 4 weeks 1 h before AlCl_3_ induction.
• **Group (6):** Rats with AlCl_3_-induced neuroinflammation were fed UF cheese supplemented with the BR complex coacervate (0.3 g/2.25 g UF/rat/d) for 4 weeks 1 h before AlCl_3_ induction.
• **Group (7):** Rats with AlCl_3_-induced neuroinflammation were fed UF cheese supplemented with the SL complex coacervate (60 mg/2.25 g UF/rat/d) for 4 weeks 1 h before AlCl_3_ induction.

During the experiment, the rats were fed a well-balanced diet. Food consumption was tracked on a daily basis. The total food intake, BW gain, and feed efficiency ratio were determined. All animal groups were starved for 12 h before being euthanized by cervical dislocation. They were sedated with ketamine hydrochloride (35 mg/kg IM) after a 4-week research period before euthanasia.

##### Biochemical Parameters

###### Preparation of Brain Homogenates.

Brain tissues were dissected from each animal, placed on ice, and stored in 10% saline. The brain tissues were homogenized in PBS (pH 7.4) and subjected to high-speed centrifugation at 4,000 rpm for 20 min (4°C). The resultant supernatant was used for the biochemical analysis. All rat brain homogenates were promptly assessed for malondialdehyde (MDA) (44, 45), superoxide dismutase (SOD) (46), CAT (47), glutathione peroxidase (GPx) (48), reduced glutathione (GSH) (49), nitric oxide (NO) (50), acetylcholinesterase (AChE) (51), and butyrylcholinesterase (52). Additionally, dopamine (DA), 5-hydroxytryptamine (5-HT; serotonin), brain-derived neurotrophic factor (BDNF), and glial fibrillary acidic protein (GFAP) levels were determined by an enzyme-linked immunosorbent assay with ELISA kits (Sunlong Biotech, China).

###### Statistical Analyses.

SPSS (IBM^®^ SPSS^®^, 2017) was used to analyze data (mean values and standard deviation, or mean values and standard error). Differences at 5% significance were assessed using ANOVA and Duncan’s multiple range tests (*P* < 0.05; *P* < 0.005). All of the tests were repeated three times, and the analysis was performed in triplicate (53).

## Results and Discussion

### High-Performance Liquid Chromatography Analysis of Phenolic Extracts

The HPLC profiles of RB, BR, and SL were analyzed for 17 phenolic compounds, including gallic acid, chlorogenic acid, methyl gallate, caffeic acid, syringic acid, pyrocatechol, rutin, ellagic acid, coumaric acid, vanillin, ferulic acid, naringenin, daidzein, quercetin, cinnamic acid, apigenin, kaempferol, and hesperetin. The quantitative results are shown in Table 1. The total amount of phenolics in the SL extract was the greatest, followed by the BR extract and the RB extract (12801.16, 4923.08, and 541.77 μg/g, respectively). For the RB extract, six major phenolics were observed with concentrations of 11.67, 252.04, 9.68, 51, 79.14, and 38.24 μg/g for gallic acid, chlorogenic acid, syringic acid, daidzein, kaempferol, and hesperetin, respectively. Although chlorogenic and gallic acids had the highest concentrations in the BR extract (1506.3 and 1241.6 g/g, respectively), the phenolic compound with the lowest concentration was caffeic acid (13.24 μg/g). From the results in Table 1, it is clear that the highest concentration of phenolic compounds in the SL extract was observed for daidzein, followed by quercetin (3984.8 and 2836.7 μg/g, respectively).

**TABLE 1:** Phenolic compound profile (μg/g) of red beet, broccoli, and spinach leaf extracts using HPLC.

Phenolic compounds conc. (μg/g)	Red beet	Broccoli	Spinach leaves
Gallic acid	111.67	1241.57	453.84
Chlorogenic acid	252.04	1506.33	646.86
Methyl gallate	0	783.47	62.88
Caffeic acid	0	13.24	100.88
Syringic acid	9.68	84.13	865.14
Pyro catechol	0	21.25	0
Rutin	0	158.55	400.82
Ellagic acid	0	167.37	1621.3
Coumaric acid	0	66.34	92.07
Vanillin	0	62.2	219.24
Ferulic acid	0	54.21	684.19
Naringenin	0	365.18	805.31
Daidzein	51	207.37	3984.79
Quercetin	0	85.65	2836.71
Apigenin	0	21.95	0
Kaempferol	79.14	0	23.21
Hesperetin	38.24	84.27	3.92
Total	**541.77**	**4923.08**	**12801.16**

### Complex Coacervate Characterization

#### ζ-Potential Analysis

**FIGURE 1:**
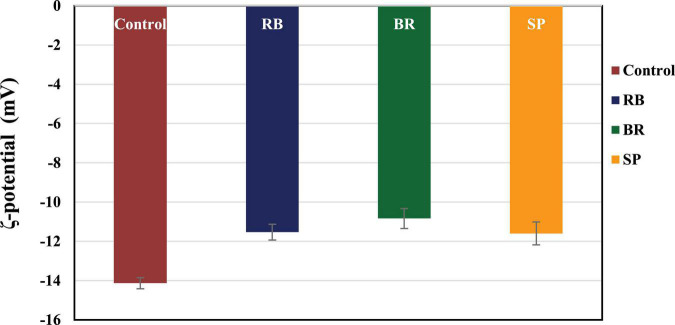
ζ-Potential of control (without phenolic extract), red beet (RB), broccoli (BR), and spinach leaf (SL) complex coacervate microcapsules.

The ζ-potential values of the RB, BR, and SL complex coacervates are shown in Figure 1. The colloidal molecules interact depending on their ζ-potential; therefore, determining the ζ-potential explains possible repulsive interactions between charged molecules (54). The RB, BR, and SL complex coacervates had –11.55 ± 0.64, –10.75 ± 0.78, and –11.90 ± 0.57 mV ζ-potentials, respectively. Because of the higher WPC and GA weights in solution, the ζ-potential of the RB, BR, and SL complex particles was always negatively charged. The RB, BR, and SL complex coacervates had ζ-potential values close to zero, indicating that electrostatic interactions neutralize the charges of biopolymers, as predicted in the complex coacervation technique (12). Aggregations were caused primarily by a shortage of electrostatic interactions among biomolecules, which resulted in the development of insoluble microcapsule coacervates (55). However, a ζ-potential of approximately 30 mV suggested a steady diffusion system because of the dominance of electrostatic interparticle interactions (56). The ζ-potential values assisted in determining the total charge of the microcapsules included in the diffusion GA/WPC, indicating charge neutralization through electrostatic interactions.

#### Encapsulation Efficiency

**FIGURE 2:**
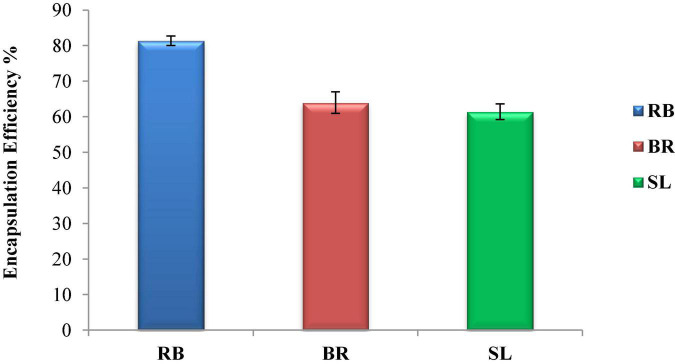
Encapsulation efficiency (EE %) of the red beet (RB), broccoli (BR), and spinach leaf (SL) complex coacervate microcapsules.

Figure 2 shows the encapsulation efficiency of the RB, BR, and SL complex coacervates. However, the greatest EE (81.3%) was reported for the RB complex coacervate, followed by the EEs of both the BR and SL complex coacervates. This is in agreement with the findings of El-Messery et al. (57), who reported that orange peel polyphenol extract was maintained and included in functional yogurt because of the encapsulation of this extract in WPC and GA. Additionally, the WPC was used for polyphenol encapsulation as an encapsulated material in oat bran biotechnology studies (58). The EE was 95.28% when WPC and MD were used at a ratio of 60:40. Furthermore, spray-drying monomeric anthocyanins in whey protein isolates, according to Flores et al. (59), resulted in an encapsulation effectiveness of 70%. Ong et al. (60) reported that anthocyanins extracted from the peel of sour cherries were successfully packaged in a whey protein isolate, with a 70.30 ± 2.20% encapsulation efficiency. Overall, encapsulation efficiency depends on the interaction between phenolics and protein to form the phenolic–protein complex. This interaction is based on strong hydrophobic and hydrogen bonds. Therefore, the red beet phenolic extract may contain a phenolic group that acts as an excellent hydrogen donor. When interacting with protein, the phenolic group will form a strong hydrogen bond with the carboxyl group in protein.

#### Morphology

**FIGURE 3:**
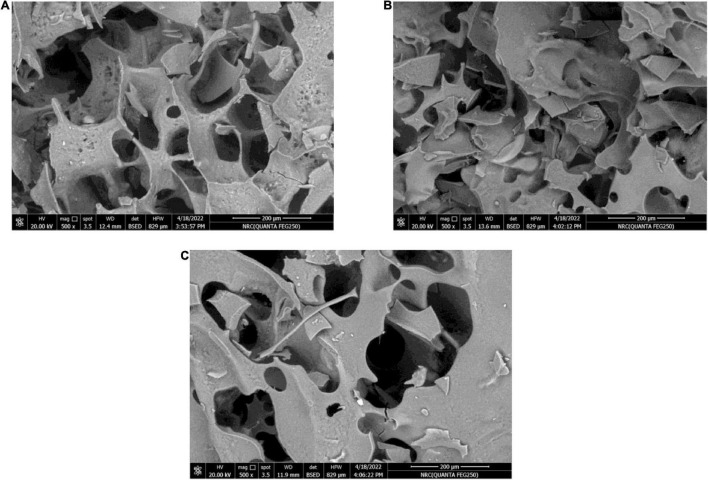
Scanning electron microscope (SEM) images of the red beet (RB) **(A)**, broccoli (BR) **(B)**, and spinach leaf (SL) **(C)** complex coacervate microcapsules (after freeze-drying).

Figure 3 demonstrates the WPC/GA ratio, which provides a protective coating for the RB, BR, and SL phenolic extracts. The particles containing the RB, BR, and SL phenolic extracts were examined by scanning electron microscopy. It was observed that the microcapsules had smoother and fewer wrinkles on the surface, presumably because of the presence of the extracts in microparticles, which decreased evaporation from the surface and increased the particle moisture content. This feature of the microcapsules was caused by the rapid sublimation of ice water from the wall component, resulting in the formation of a hollow in a crystallite surface without enough time for wrinkles to form (61). These findings demonstrate that using a WPC/GA ratio of 60:40 as the wall material can microencapsulate phenolics with the lowest particle diameter (321–338 nm), which is consistent with previous findings (57, 58, 62).

#### FTIR Spectroscopy

**FIGURE 4:**
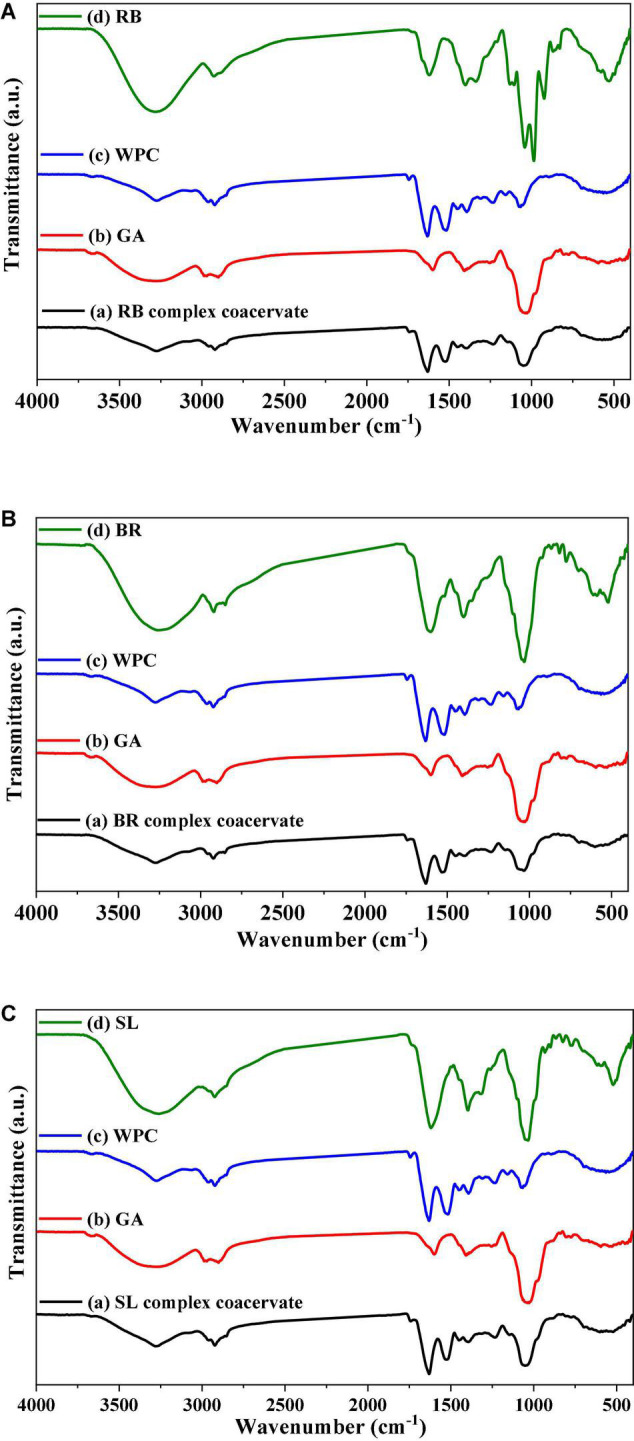
FTIR spectrum of the red beet (RB) **(A)**, broccoli (BR) **(B)**, and spinach leaf (SL) **(C)** powdered extracts encapsulated with different wall materials. WPC, whey protein concentrate; GA, gum arabic.

The FTIR spectra of the powdered extract, its microcapsules (RB, BR, and SL), and the raw materials utilized to make the microcapsules in this investigation are presented in Figure 4. These formulations were studied in their solid phases to avoid the influence of water absorption. In the FTIR spectrum of pure GA, OH stretching, which is typical of the glycosidic ring vibration of hydroxyl groups, contributed to the presence of a broad band at 3,274 cm^–1^, whereas GA revealed a signal for the dilation of C–H at 2,971 cm^–1^. Furthermore, the COO-symmetric stretching of GA yielded a prominent signal at 1,637 cm^–1^. The peaks at 1,454 cm**^–1^** were related to the asymmetric dilation of COO–, whereas the peaks at 1,254–1,029 cm^–1^ were the fingerprint of carbohydrates. However, the FTIR spectrum of WPC was 1,648 and 1,453 cm^–1^. The peaks of the phenolic OH groups were at 3,258, 3,279, and 2,359 cm^–1^ in the raw material FTIR spectra of pure BR, RB, and SL powdered extracts, respectively. The other phenolic compounds from the extracts were capped on the surface of the produced nanoparticles, stabilizing them, as shown by the presence of the other distinctive peaks of extracts in the FTIR spectrum of the WPC. There were significant changes in peak positions in the bands because of hydrogen bonding between phenolics and proteins. The interactions of WPC with several monophenolic, diphenolic, and polyphenolic chemicals are clarified in Figures 4A–C (57). The interaction between phenolics in WPC was studied using FTIR, which revealed the formation of certain complex hydrogen bonds. Furthermore, electrostatic interactions are important in the stabilization of the peptide by phenolic compounds. Phenolic compounds were strategically placed near the peptide side chain groups. The active groups para-OH (p-OH), meta-OH (m-OH), and COOH of phenolics are postulated to act as hydrogen bond donors/acceptors for various amino acid side chain groups (63).

#### Chemical Composition of Cheese

Table 2 shows the chemical composition of cheese fortified with the RB, BR, and SL extract complex coacervates on the day of processing. As shown in Table 2, there was no significant variation in composition (*P* < 0.05) of free complex coacervates of cheese and the fortified cheese samples of the RB, BR, and SL complex coacervates (except for pH). This implies that, in this experiment, fortifying cheese with the RB, BR, and SL complex coacervates did not negatively influence the chemical analysis of UF cheese. As indicated in Table 2, there was a minor change in pH values between the control cheese and cheese fortified with the RB, BR, and SL complex coacervates. The control cheese had the highest pH, whereas the cheese fortified with the BR complex coacervate had the lowest pH. Adding tomato extract microcapsules to Queso Blanco cheese caused a comparable decrease in cheese pH in the study by Jeong et al. (64). The acidic pH of these compounds may explain the lowering of the pH of the medium to which bioactive compounds were introduced. This finding is similar to the findings of the HPLC analysis for the RB, BR, and SL extracts, which showed that the BR extract had approximately three times the phenolic content of the SL extract and nine times that of the RB extract.

**TABLE 2:** Chemical analysis of UF cheese with the RB, BR, and SL complex coacervates.

Parameters	Control	RB	BR	SL
**Moisture, %**	69.17 ± 0.34^a^	68.53 ± 0.40^a^	68.52 ± 0.38^a^	68.52 ± 0.42^a^
**Total solids, %**	30.83 ± 0.34^a^	31.48 ± 0.40^a^	31.48 ± 0.38^a^	31.49 ± 0.42^a^
**Fat, %**	10.53 ± 0.04^a^	10.54 ± 0.02^a^	10.54 ± 0.01^a^	10.53 ± 0.03^a^
**Protein, %**	10.95 ± 0.14^a^	11.30 ± 0.11^a^	11.32 ± 0.16^a^	11.32 ± 0.13^a^
**pH**	6.73 ± 0.02^a^	6.64 ± 0.01^b^	6.62 ± 0.01^c^	6.68 ± 0.01^ab^

*All the values are the mean ± Stdv. ^a,b,c^Within rows, the means with different letters were significantly different (P < 0.05). Control: UF cheese without a complex coacervate; RB: UF cheese with a red beet complex coacervate; BR: UF cheese with a broccoli complex coacervate; and SL: UF cheese with a spinach leaf complex coacervate.*

#### Texture Analysis

Texture profile analysis (TPA) is a useful measure of the textural quality of a cheese product that corresponds well with sensory characteristics (60). The texture profile analysis of all cheese samples was performed in this study for chewiness, hardness, cohesiveness, gumminess, and adhesiveness, as provided in Table 3. The chewiness, hardness, cohesiveness, gumminess, and adhesiveness of the fortified UF cheese containing the RB, BR, and SL extract complex coacervates differed slightly (*P* < 0.05), as seen in Table 3. Ionic entities crosslinked by covalent bonds to casein strands were protonated during cheese formation when the pH dropped. As a result, the connection between casein micelles strengthened, forming tough curds (65). In this respect, Ong et al. (60) found that curds manufactured with rennin at pH 6.5 were tougher than curds manufactured with renin at pH 6.1. This finding was also true in the present investigation, in which the pH of the UF cheese samples fortified with RB, BR, and SL complex coacervates was higher than that of the control cheese. As a result, the presence of the RB, BR, and SL complex coacervates in the current investigation could be one of the factors contributing to the increase in hardness, cohesiveness, and gumminess of the fortified cheese (BR > SL > RB).

**TABLE 3:** Texture parameters of UF cheese with the RB, BR, and SL complex coacervates.

Samples	Hardness (N)	Springiness (mm)	Cohesiveness	Gumminess (N)	Chewiness (N*mm)
**Control**	10.405 ± 0.19^c^	0.76 ± 0.05^a^	0.65 ± 0.01^c^	6.68 ± 0.07^c^	5.14 ± 0.49^a^
**RB**	11.56 ± 0.25^b^	0.82 ± 0.04^a^	0.74 ± 0.01^ab^	7.26 ± 0.11^b^	5.22 ± 0.35^a^
**BR**	12.73 ± 0.60^a^	0.77 ± 0.02^a^	0.73 ± 0.01^b^	8.36 ± 0.11^a^	6.12 ± 0.42^a^
**SL**	12.02 ± 0.13^ab^	0.77 ± 0.03^a^	0.76 ± 0.01^a^	6.93 ± 0.13^c^	6.09 ± 0.28^a^

*All the values are the mean ± Stdv. ^a, b, c^Within a column, the means with different letters were significantly different (P < 0.05). Control: UF cheese without a complex coacervate; RB: UF cheese with a red beet complex coacervate; BR: UF cheese with a broccoli complex coacervate; and SL: UF cheese with a spinach leaf complex coacervate.*

#### Sensory Evaluation

Sensory evaluation of cheese is crucial to the feasibility of an industrial manufacturing approach to food organoleptic properties (66). The total sensory scores of UF cheese comprise color, appearance, body, texture, and flavor, as illustrated in Table 4. The sensory score of UF cheese fortified with the RB, BR, and SL extract complex coacervates was somewhat higher than that of the control cheese. These results could be attributed to the favorable impact of RB, BR, and SL extract complex coacervate fortification on the UF cheese compared to the control cheese; they enhanced the flavor and appearance scores. These findings are consistent with those of our previous study (57), in which we found that fortifying processed cheese with a phenolic extract from the mandarin peel in the form of liposomes had little influence on sensory characteristics, which remained acceptable. The current findings are congruent with those of Farrag et al. (67). Cheese supplemented with polyphenol capsules had higher overall rankings and improved flavor, body, texture, and acceptance.

**TABLE 4:** Sensory characteristics of UF cheese fortified with the RB, BR, and SL complex coacervates.

Samples	Color and appearance	Body and texture	Flavor
**Control**	18.50 ± 1.00^a^	43.17 ± 1.26^a^	32.00 ± 1.50^a^
**RB**	14.67 ± 1.53^b^	44.00 ± 1.00^a^	26.50 ± 1.80^b^
**BR**	14.00 ± 2.00^b^	40.00 ± 2.00^b^	21.33 ± 1.53^c^
**SL**	14.00 ± 2.00^b^	44.00 ± 1.00^a^	20.00 ± 1.00^c^

*All the values are the mean ± Stdv. ^a, b, c^Within the same column, the means with different letters were significantly different (P < 0.05). Control: UF cheese without a complex coacervate; RB: UF cheese with a red beet complex coacervate; BR: UF cheese with a broccoli complex coacervate; and SL: UF cheese with a spinach leaf complex coacervate.*

#### Nutritional Parameters

Table 5 shows the initial and final body weights, body weight gain, total food intake, and food efficiency of the negative control, AlCl_3_-induced neuroinflammation (positive control), AlCl_3_-induced neuroinflammation + UF cheese (gp. 4), and all treated groups (groups 1, 2, 3, 5, 6, and 7), which were examined in a 4-week trial. There were no significant variations (*P* > 0.05) in initial body weights; total food intake and food efficiency did not differ in all treated groups, including RB, BR, and SL extracts; and UF cheese containing the RB, BR, and SL complex coacervates represented as 1, 2, 3, 5, 6, and 7 when compared to the AlCl_3_-induced neuroinflammation (positive control) and AlCl_3_-induced neuroinflammation + UF cheese (gp. 4) groups. The same result was observed in the AlCl_3_-induced neuroinflammation + UF cheese group (gp. 4) compared to the AlCl_3_-induced (positive) group, and the AlCl_3_-induced (positive) group compared to the negative control group. This result agreed with that of several studies (68, 69). In contrast, a significant reduction (*P* ≤ 0.05) in body weight was detected in the AlCl_3_-induced neuroinflammation (positive) and AlCl_3_-induced neuroinflammation + UF cheese (gp. 4) groups compared to the negative control (70). A significant elevation in body weight occurred in all treated groups (1, 2, 3, 5, 6, and 7) compared to the AlCl_3_-induced neuroinflammation (positive) and AlCl_3_-induced neuroinflammation + UF cheese (gp. 4) groups.

**TABLE 5:** Effects of the RB, BR, and SL complex coacervates and UF cheese with coacervates on body weight, body weight gain, total food intake, and food efficiency.

Groups	Initial body weight (g)	Final body weight (g)	Body weight gain (g)	Total food intake (g)	Food efficiency*
**Negative control**	205.2 ± 2.65	253.7 ± 3.18	48.5 ± 0.53	3536.5 ± 2.22	0.014 ± 0.24
**Positive control**	207.3 ± 3.21^a^	244.9 ± 4.06^a^	37.6 ± 0.85^a^	3525.5 ± 2.15^a^	0.011 ± 0.4^a^
**Group (1)**	206.2 ± 3.37^b^	249 ± 3.91^b^	42.8 ± 0.54^b^	3533.5 ± 2.31^b^	0.012 ± 0.23^b^
**Group (2)**	206.1 ± 3.43^b^	249.5 ± 3.9^c^	43.4 ± 0.47^c^	3531.4 ± 2.33^b^	0.012 ± 0.2^b^
**Group (3)**	205.2 ± 3.53^b^	251.9 ± 4.31^d^	46.7 ± 0.78^d^	3534.6 ± 2.41^b^	0.013 ± 0.32^b^
**Group (4)**	204.9 ± 3.25^ab^	243.6 ± 3.78^ab^	38.7 ± 0.53^ab^	3525.5 ± 2.27^ab^	0.011 ± 0.23^ab^
**Group (5)**	205.1 ± 3.12^b^	247.9 ± 3.57^e^	42.8 ± 0.45^e^	3533.4 ± 2.32^b^	0.012 ± 0.19^b^
**Group (6)**	204.2 ± 3.63^b^	247.8 ± 4.36^f^	43.6 ± 0.73^f^	3531.2 ± 2.43^b^	0.012 ± 0.3^b^
**Group (7)**	204.9 ± 3.55^b^	251.3 ± 4.11^g^	46.4 ± 0.56^g^	3534.5 ± 2.28^b^	0.013 ± 0.25^b^

Values are the mean ± SE (n = 6). The same letters in each column reflect a non-significant difference across treatments, whereas different letters reflect a significant difference (P ≤ 0.05, P ≤ 0.005). *Food efficiency, body weight gain/total food intake.

#### Biochemical Parameters

AlCl_3_ is toxic and enters the food chain through drinking water and food (71). It crosses the blood–brain barrier (BBB) and aggregates in the brain, mainly in the hippocampus, which is responsible for memory and cognition. AlCl_3_ induced biochemical changes and oxidative stress in a rat brain model and may evoke neuronal disorders in human. Aluminum accumulates in the brain over time, causing neuroinflammation in the form of neurofibrillary tangles and amyloid aggregates. As a result, elucidating the specific mechanism of AlCl_3_-induced neuroinflammation is critical. This prompted us to investigate the impact of the RB, BR, and SL extracts and UF cheese containing complex coacervates of the RB, BR, and SL extracts in the AlCl_3_-induced rat model.

Acetylcholine is a cholinergic neurotransmitter that is controlled by acetylcholinesterases (AChE and BChE), the main enzymes that break down acetylcholine. In Table 6, it is shown that AlCl_3_ administration to the rats led to a significant elevation in AChE and BChE in AlCl_3_-induced neuroinflammation (positive) and AlCl_3_-induced neuroinflammation + UF cheese groups compared to the negative control (72). AlCl_3_ can trigger an imbalance in neurotransmitter levels (73). Additionally, the rats treated with the RB, BR, and SL extracts showed a significant reduction similar to rats treated with UF cheese containing complex coacervates of the RB, BR, and SL extracts in AlCl_3_-treated rats and suppressed AChE and BChE activities. This result is consistent with that of previous studies. Similarly, the mode of action of the RB, BR, and SL extracts can be utilized in the treatment of neurodegenerative diseases and memory-deficit disorders, with benefits attributed to their modulatory impacts on acetylcholinesterase action, which can enhance learning and memory (74, 75). These extracts are considered the most promising in improving the neuronal damage and memory dysfunction triggered by AlCl_3_ injection, along with the advancement of cholinergic activity in the brain tissue and cholinergic neurotransmission in rats with memory impairment (76–78).

**TABLE 6:** Effects of the RB, BR, and SL complex coacervates and UF cheese with these complex coacervates on biochemical parameters.

Groups	Acetylcholinesterase (ng/g tissue)	Butyrylcholinesterase (U/g tissue)	Dopamine (μ g/g tissue)	5-hydroxytryptamine (μ g/g tissue)	BDNF (pg/g tissue)	GFAP (pg/g tissue)
**Negative control**	0.99 ± 0.08	310.64 ± 7.42	449.64 ± 2.12	44.34 ± 1.32	24.13 ± 0.34	243.34 ± 1.67
**Positive control**	2.34 ± 0.06^a^	611.32 ± 8.13^a^	231.42 ± 2.33^a^	27.32 ± 1.43^a^	11.19 ± 0.23^a^	539.89 ± 1.92^a^
**Group (1)**	1.76 ± 0.07^b^	405.43 ± 15.04^b^	325.13 ± 2.14^b^	32.23 ± 1.19^b^	17.45 ± 0.19^b^	315.63 ± 1.34^b^
**Group (2)**	1.45 ± 0.07^c^	402.32 ± 16.11^c^	332.35 ± 2.16^c^	33.39 ± 1.21^c^	18.21 ± 0.23^c^	302.39 ± 1.26^c^
**Group (3)**	1.38 ± 0.07^d^	398.43 ± 13.25^d^	395.42 ± 2.21^d^	39.48 ± 1.16^d^	21.13 ± 0.16^d^	296.22 ± 1.21^d^
**Group (4)**	2.29 ± 0.06^a^	602.12 ± 8.18^a^	235.49 ± 2.43^a^	27.12 ± 1.37^a^	12.08 ± 0.22^a^	535.73 ± 1.84^a^
**Group (5)**	1.74 ± 0.07^e^	403.23 ± 14.64^e^	322.14 ± 2.11^e^	31.53 ± 1.29^e^	17.12 ± 0.19^e^	311.23 ± 1.32^e^
**Group (6)**	1.43 ± 0.07^f^	401.32 ± 15.71^f^	327.31 ± 2.12^f^	32.67 ± 1.21^f^	18.11 ± 0.23^f^	300.67 ± 1.27^f^
**Group (7)**	1.34 ± 0.07^g^	396.33 ± 13.68^g^	390.37 ± 2.17^g^	37.38 ± 1.16^g^	20.97 ± 0.16^g^	299.61 ± 1.23^g^

*Values are represented as the mean ± SE (n = 6). The same letter in each column reflects a non-significant difference across treatments, whereas different letters reflect a significant difference (P ≤ 0.05, P ≤ 0.005).*

As seen in Table 6, the brain (hippocampus and cortex homogenate) neurotransmitters (5-HT and DA) significantly decreased in the AlCl_3_-induced neuroinflammation (positive) and AlCl_3_-induced neuroinflammation + UF cheese groups (gp. 4) compared to the negative control. This could be the reason for the development of some neurodegenerative and neuropsychiatric diseases. However, the administration of the RB, BR, and SL extracts and UF cheese containing complex coacervates of the RB, BR, and SL extracts significantly elevated these neurotransmitters compared to the AlCl_3_-induced neuroinflammation (positive) and AlCl_3_-induced neuroinflammation + UF cheese groups. The preventive effect of the RB, BR, and SL extracts against the depletion of neurotransmitters is caused by their polyphenolic and flavonoid contents, which are involved in elevating brain dopamine levels by suppressing monoamine oxidase-B action, which is effective in avoiding aging-associated cognitive impairments. BDNF plays a vital role in nerve cell survival and synaptic plasticity and participates in a number of neurological deficits (79). GFAP is the major filament in the astrocytic cytoskeleton and has long been utilized as a biomarker for neurotoxicity and astrocyte activation (80). The results in Table 6 demonstrate that there was a significant decrease in BDNF and a significant increase in GFAP levels in both the AlCl_3_-induced neuroinflammation (positive) and AlCl_3_-induced neuroinflammation + UF cheese groups compared to the negative control group. The RB, BR, and SL extracts and UF cheese containing complex coacervates of the RB, BR, and SL extracts exhibited a significant elevation in BDNF and a significant reduction in GFAP levels compared to AlCl_3_-induced neuroinflammation (positive control) and AlCl_3_-induced neuroinflammation + UF cheese groups. In the central nervous system, flavonoids target astrocytes and promote BDNF release while inhibiting GFAP expression (81). This may explain the ability of the RB, BR, and SL extracts to elevate BDNF and reduce GFAP levels following AlCl_3_ exposure in our present research.

The brain is a susceptible organ to oxidative stress owing to its high oxygen consumption, low mitotic rate, and low antioxidant levels. Neuronal injury is caused by extensive oxidative damage caused by AlCl_3_ toxicity, which disrupts the antioxidant defense mechanism of the brain (82) by promoting the buildup of reactive oxygen species in cells that can eventually result in the elevated expression of genes encoding antioxidant enzymes (83).

SOD is a pioneering antioxidant enzyme that neutralizes singlet oxygen and spontaneously dismutates superoxide radicals to hydrogen peroxide. CAT is a naturally produced cellular antioxidant that is responsible for reducing oxidative stress. CAT effectively decomposes hydrogen peroxide and therefore avoids lipid peroxidation (84). GPx enhances hydrogen peroxide and lipid peroxide reduction, whereas GR stimulates the NADPH-driven conversion of GSSG to GSH (85). The reduction among these antioxidant enzymes and molecules may be linked to an excessive buildup of hydrogen peroxide, which inhibits neuronal antioxidant defenses (86). AlCl_3_ reduced the activities of GPx, SOD, and GR. Both MDA as a lipid peroxidation marker and NO as an inflammatory marker in the brain (cerebrum, cerebellum, and midbrain) are essential for neuronal cells that are considerably susceptible to oxidative stress and inflammation; thus, they require an effectual steady supply of powerful antioxidants to neutralize the lipid peroxidation chain reaction that causes free radical destruction (87).

According to Table 7, AlCl_3_ administration caused oxidative stress, which was reflected in a significant increase in CAT, MDA, and NO and a significant reduction in SOD, glutathione peroxidase, and glutathione in both the AlCl_3_-induced neuroinflammation (positive) and AlCl_3_-induced neuroinflammation + UF cheese groups in comparison with the negative control. There is a disturbance in redox homeostasis and an imbalance between oxidative stress and antioxidant defenses that occur when free radicals accumulate excessively in the body, causing damage to biological macromolecules, which leads to brain damage and severe cognitive impairment (88–91). The RB, BR, and SL extracts and UF cheese containing complex coacervates of the RB, BR, and SL extracts induced a significant elevation of SOD and glutathione peroxidase and reduced glutathione with a significant reduction in the CAT, MDA, and NO levels compared to AlCl_3_-induced neuroinflammation (positive) and AlCl_3_-induced neuroinflammation + UF cheese groups. The results in Table 7 are compatible with those of prior studies that revealed that the administration of RB, BR, and SL extracts and UF cheese containing complex coacervates of the RB, BR, and SL extracts was linked to the prevention of oxidative reactions *via* prooxidant inhibition and endogenous antioxidant system activation (92). This prevents tissue damage by inhibiting enzyme leakage *via* neuronal cell membranes (79) in rats. A potential process between the lowered enzyme activities examined and the effect of RB, BR, and SL extracts could be a synergetic effect between the antioxidant activity of the phenolic and flavonoid compounds and endogenous antioxidants, which maintains redox homeostasis after AlCl_3_ administration (93). These findings may explain why RB, BR, and SL treatments mitigated the overall severity of rat brain tissue damage, demonstrating their therapeutic effects against AlCl_3_-induced impairments (94). Hence, the antioxidant activity of RB, BR, and SL as neuroprotective drugs against AlCl_3_-induced neuroinflammation in rat models was further supported by our findings.

**TABLE 7:** Effects of the RB, BR, and SL complex coacervates and UF cheese with complex coacervates on biochemical parameters.

Groups	Superoxide Dismutase (U/g tissue)	Catalase (μ mol/g tissue)	Glutathione peroxidase (U/g tissue)	Reduced glutathione (μ mol/g tissue)	Malonaldehyde (nmol/g tissue)	Nitric oxide (nmol/g tissue)
**Negative control**	7.48 ± 0.49	3.63 ± 0.24	4.84 ± 0.74	13.56 ± 0.78	5.23 ± 0.39	1.99 ± 0.29
**Positive control**	4.19 ± 0.33^a^	7.82 ± 0.42^a^	3.32 ± 0.64^a^	9.89 ± 0.49^a^	14.97 ± 0.81^a^	3.89 ± 0.23^a^
**Group (1)**	5.41 ± 0.35^b^	4.32 ± 0.32^b^	4.12 ± 0.72^b^	10.82 ± 0.32^b^	11.21 ± 0.72^b^	2.41 ± 0.22^b^
**Group (2)**	6.48 ± 0.28^c^	4.21 ± 0.28^c^	4.33 ± 0.68^c^	11.71 ± 0.58^c^	10.43 ± 0.69^c^	2.38 ± 0.21^c^
**Group (3)**	6.93 ± 0.27^d^	3.82 ± 0.31^d^	4.62 ± 0.71^d^	12.98 ± 0.51^d^	9.49 ± 0.61^d^	2.11 ± 0.19^d^
**Group (4)**	4.23 ± 0.33^a^	7.63 ± 0.41^a^	3.41 ± 0.65^a^	9.76 ± 0.48^a^	13.86 ± 0.82^a^	3.65 ± 0.24^a^
**Group (5)**	5.42 ± 0.34^e^	4.31 ± 0.33^e^	4.07 ± 0.71^e^	10.75 ± 0.31^e^	11.55 ± 0.74^e^	2.52 ± 0.23^e^
**Group (6)**	6.43 ± 0.28^f^	4.19 ± 0.29^f^	4.26 ± 0.67^f^	11.63 ± 0.54^f^	10.62 ± 0.64^f^	2.46 ± 0.22^f^
**Group (7)**	6.91 ± 0.27^g^	3.93 ± 0.34^g^	4.58 ± 0.71^g^	12.72 ± 0.51^g^	9.51 ± 0.67^g^	2.19 ± 0.19^g^

*Values are represented as the mean ± SE (n = 6). The same letters in each column reflect a non-significant difference across treatments, whereas different letters reflect a significant difference (P ≤ 0.05).*

**FIGURE 5:**
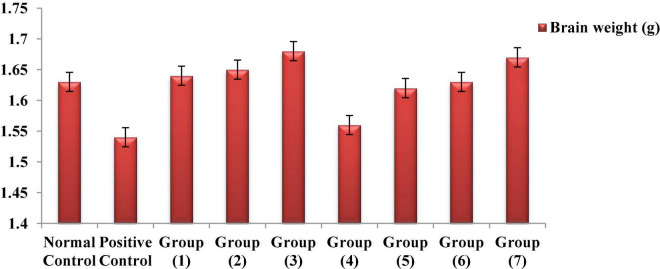
Effects of the RB, BR, and SL complex microcapsules and UF cheese with complex coacervates on the brain weight of rats.

Numerous study results have shown that, if chemical toxins are not treated, they cause significant brain weight loss in rats (95–97). According to Figure 5, the observed reduction results for the brain weights of both the AlCl_3_-induced neuroinflammation and AlCl_3_-induced neuroinflammation + UF cheese groups compared to the negative control, which could have occurred because of nutrient depletion, modification of physiological functions or pathways, and protein denaturation (97). The administration of the RB, BR, and SL extracts and UF cheese containing complex coacervates of the RB, BR, and SL extracts significantly elevated the brain weight of rats to the normal level because of their polyphenolic and flavonoid contents. This could lead to a significant reduction in the oxidative markers caused by both antioxidant and anti-inflammatory properties (98). The overall findings confirmed the ability of the RB, BR, and SL extracts and UF cheese containing the RB, BR, and SL complex coacervates to improve metabolic and neurobehavioral strategies and enhance the protein biosynthesis of the brain (99).

## Conclusion

Based on our findings, the complex coacervation technique is an excellent alternative for encapsulating the RB, BR, and SL phenolic extracts. Although the addition of the encapsulated phenolic extract complex coacervates to UF cheese decreased the pH, it did not considerably influence the composition and texture profile of the UF cheese. UF cheese with encapsulated phenolic extracts improved metabolic activities, neurobehavioral function, and protein synthesis in the brain through mechanisms that contribute to its antioxidant property, acetylcholinesterase suppression, lipid peroxidation inhibition, and anti-inflammatory and neurobehavioral restorative properties mediated by the regulation of AChE activity. Additionally, it increased BDNF, decreased GFAP, inhibited prooxidant (NO), and enhanced the cortical endogenous antioxidant substances (GSH, GPx, GR, SOD, and CAT). These findings support the use of encapsulated phenolic extracts from the RB, BR, and SL complex coacervates against AlCl_3_-induced neuroinflammation in rats. The efficient production of functional UF cheese, including encapsulated phenolic extract complex coacervates, revealed in this research could be of interest to both food producers and academics seeking to provide natural bioactive substances in the most suitable way (i.e., food).

## Data Availability Statement

The original contributions presented in this study are included in the article/supplementary material, further inquiries can be directed to the corresponding author/s.

## Ethics Statement

The animal study was reviewed and approved by the Ethical Committee of Medical Research at Egypt’s National Research Centre.

## Author Contributions

TS: visualization, methodology, and writing. DM: conceptuali-zation, formal analysis, methodology, interpretation of biological data, and writing—software review and editing. TE-M: conceptualization, methodology, and data curation. ME: methodology and performing the experiments. AZ: methodology, validation, and writing—review and editing. J-BE and J-HS: validation and writing—review and editing. ME-S: conceptualization, formal analysis, writing—original draft, methodology, data curation, and software. All authors contributed to the article and approved the submitted version.

## Conflict of Interest

The authors declare that the research was conducted in the absence of any commercial or financial relationships that could be construed as a potential conflict of interest.

## Publisher’s Note

All claims expressed in this article are solely those of the authors and do not necessarily represent those of their affiliated organizations, or those of the publisher, the editors and the reviewers. Any product that may be evaluated in this article, or claim that may be made by its manufacturer, is not guaranteed or endorsed by the publisher.
